# Diabetes related distress is high in inpatients with diabetes

**DOI:** 10.1186/s13098-021-00659-y

**Published:** 2021-04-09

**Authors:** Nadine Kuniss, Guido Kramer, Ulrich A. Müller, Gunter Wolf, Christof Kloos

**Affiliations:** 1 Department of Internal Medicine III, Endocrinology and Metabolic Disorders, Jena University Hospital, Am Klinikum 1, 07747 Jena, Germany; 2 Outpatient Healthcare Centre Dr. med. Kielstein, Erfurt, Germany

**Keywords:** Diabetes-related distress, PAID questionnaire, Psychosocial burden

## Abstract

**Objective:**

The aim of the present study was to assess diabetes-related distress in inpatients and its association with metabolic control in people with diabetes type 1 (DM1) and type 2 (DM2).

**Research design and methods:**

In a cross-sectional study, 107 inpatients with DM1 (age 45.9 years, diabetes duration 18.7 years, HbA1c 8.4%/67.8 mmol/mol) and 109 with DM2 (age 62.0 years, diabetes duration 16.2 years, HbA1c 8.9%/74.3 mmol/mol) from a University department for endocrinology and metabolic diseases (Germany) were included over 2 years. Diabetes-related distress was assessed with the PAID questionnaire (range 0–100, higher scores imply higher diabetes-related distress, cut-off ≥ 40). The PAID questionnaire was completed by 214 of 216 participants.

**Results:**

Fifty-one of 214 individuals (23.8%) showed high distress (PAID score ≥ 40). The mean PAID score was 28.1 ± 17.5 in all participants with no difference between DM1 and DM2 (28.1 ± 17.4 vs. 26.2 ± 16.9, p = 0.532). Individuals with DM2 on insulin scored higher than patients without insulin (27.8 ± 17.6 vs. 18.7 ± 8.5, p = 0.004). Additionally, people with DM1 treated with a system for continuous glucose monitoring (n = 50, 33.1 ± 18.8) scored higher than participants without such system (n = 32, 20.6 ± 13.3, p = 0.001). HbA1c was not correlated with the PAID score in both, DM1 (r = 0.040, p = 0.684) and DM2 (r = − 0.024, p = 0.804). Participants with DM2 and severe hypoglycaemia/last 12 months scored higher than people without (PAID score 43.0 ± 20.4 vs. 25.1 ± 16.5, p = 0.026). Frequency of non-severe hypoglycaemia was not associated with the PAID score in DM1 and DM2.

**Conclusions:**

Patients with diabetes treated in hospital for problems with diabetes suffer frequently from diabetes-related distress (~ 24%) regardless of diabetes type.

## Introduction

Diabetes-related distress and depression are important psychosocial factors within diabetes therapy, but should be considered as separate medical conditions. Diabetes-related stress can be defined as the lack of psychosocial adaptation to the challenges of diabetes therapy [1]. In contrast, depression can be characterised by sadness, disinterest, low self-esteem, sleep disturbances, fatigue and poor concentration. Diabetes is not an etiologically relevant factor for the diagnosis of depression. High diabetes-related distress is associated with poorer treatment adherence [2, 3], which may be the cause for poorer glycaemic control [2, 4]. Patients with high diabetes-related distress are more often female and more common type 1 diabetes, are younger and had higher BMI [3, 5]. Furthermore, diabetes-related distress is associated with a higher rate of depression [6, 7]. Depression in turn can severely impact the patients’ quality of life. The German Guideline “Psychosocial Aspects of Diabetes” reports increased incidence of depressive symptoms in people with diabetes whereby quality of life and satisfaction with treatment is reduced [1].

Thus, national and international guidelines recommend to screen regularly for psychosocial problems [1, 8]. To detect diabetes-related distress, the “Problem Area In Diabetes” (PAID) questionnaire provides a valid and reliable tool to assess emotional distress associated with diabetes [6, 9, 10]. Furthermore, the “Well-Being Index” (WHO5 questionnaire) is used to check current wellbeing [11]. Low level of emotional wellbeing indicates possible depressive disorder.

Thirty percent of people with diabetes in a German inpatient diabetes center suffered from diabetes-related distress [7]. However, there are other studies showing a lower prevalence of diabetes-related distress [12]. Prior to this study, we conducted two studies in people with diabetes type 1 (DM1) and type 2 (DM2) in an outpatient setting on primary as well as secondary care level [5, 13]. The studies showed a low prevalence of diabetes-related distress of 1.2% on primary and 8.9% on secondary level, respectively. Consequently, the prevalence of diabetes-related distress seems to vary widely and thus, the results inconclusive. However, national and international guidelines do not distinguish between care levels. It is suspected that the prevalence is even higher in hospitalised patients.

Therefore, the primary aim of the present study was to assess diabetes-related distress in an inpatient setting and to investigate its association with metabolic control in people with DM1 and DM2. Secondary, we assessed quality of life, treatment satisfaction as well as wellbeing.

## Research design and methods

### Participants

Two hundred and sixteen people with diabetes (107 with DM1 and 109 with DM2) from a large University inpatient department of endocrinology and metabolic diseases were included in this trial in the period from 07/2017 to 06/2019. All patients who have been treated in hospital for diabetes-related problems during the investigation period were interviewed. Patients with the diagnosis of impaired glucose tolerance or newly diagnosed diabetes (diabetes duration ≤ 4 weeks), people with insufficient understanding of German language and pregnant women were excluded.

### Questionnaires

We used the PAID questionnaire (20 items) to assess diabetes-related distress [10]. The PAID questionnaire is a reliable and valid tool resulting in a Cronbachʼs α of 0.95. The items of the PAID questionnaire focus on different problem areas of diabetes (e.g., fear of hypoglycaemia or long-term complications, dissatisfaction with support of family or diabetes physician, see Table 2). Each item of the PAID questionnaire is scored by values from 0 (“no problem”) to 4 (“serious problem”). All 20 scores are added up and multiplied by 1.25 resulted in a total score 0–100 points. Higher scores indicate more diabetes-related distress (cut-off ≥ 40 indicates high distress) [6, 7, 9, 10]. In addition, we analysed the 20 items separately to rank the problem areas.

Furthermore, we used three additional questionnaires: WHO5 questionnaire (Cronbachʼs α of 0.85–0.93) to assess current wellbeing [11], “Audit of Diabetes Dependent Quality of Life” questionnaire (ADDQoL, Cronbachʼs α of 0.85) to assess quality of life [14], “Diabetes Treatment Satisfaction Questionnaire status” questionnaire (DTSQs, German version of the DTSQ: Cronbachʼs α of 0.81 [15]) to assess treatment satisfaction [16].

The WHO-5 questionnaire consists of five questions (each item: 0–5 points, total score: 0–25). Higher scores are associated with higher well-being (cut off < 13 points indicates a possible depression). The ADDQoL score ranged from − 9 to + 3 (higher scores are associated with higher diabetes-related quality of life). DTSQs score ranges from 0 to 36 (higher scores indicate greater treatment satisfaction).

### Parameters

Laboratory and clinical data were drawn from our digital patient record (called “EMIL”) [17] and were collected on the day of the survey of the respective participant.

Non-severe hypoglycaemia was defined if typical symptoms (e.g. sweating, weak concentration, feeling shaky) were present but disappeared quickly after carbohydrate intake or a plasma glucose ≤ 3.9 mmol/l without typical symptoms [18]. Severe hypoglycaemia was defined as a condition with necessity of glucagon injection (administered by third party, e.g. relatives) or intravenous glucose injection (administered by medical professionals) with or without hospitalisation according to the guidelines of the German Diabetes Association [19].

HbA1c was measured using high-performance liquid chromatography (TOSOH-Glykohaemoglobin-Analyzer HLC-723 GhbV, Tosoh Corporation, Tokyo, Japan) with a normal range of 5.0–6.2%. Additionally, to compare HbA1c values with other studies, HbA1c was adjusted according to the mean normal value of healthy people (5.05%, 32 mmol/mol) according to the DCC trial [20].

We collect laboratory and clinical data (e.g. age, HbA1c, insulin dosage) of each patient within the inpatient admission to our hospital (routine procedure). In addition, the PAID-, ADDQoL-, DTSQs as well as WHO-5 questionnaire are given to each inpatient to check for psychological problems. No additional questionnaires or tests were performed in addition to routine procedure. Therefore, approval by ethical committee was not necessary for this database analysis. “Non-responders” are defined as inpatients who refused to fill in the questionnaires (only laboratory and clinical data were available). All data are analysed pseudonymously.

### Statistical analyses

Statistical analyses were performed with SPSS 25 (IBM Corporation, Armonk, NY, USA). All continuous data are presented as mean ± standard deviation (SD). Categorical data are described by absolute and relative frequencies. An unpaired *t*-test was used for continuous variables to compare two groups. Fisher’s exact test was performed for categorical variables. Pearson’s and Spearman’s correlation coefficient was calculated for assessing relationship between two variables. Significance was defined at the 0.05 level.

## Results

Two hundred ninety-one people were invited to participate in the study. Seventy-five people (25.8%) refused to take part. Thus, a total of 216 people were included in the study. Baseline characteristic of the study participants and non-responders are shown in Table 1. There were differences between the included patients and the non-responders regarding gender, age, diabetes therapy, self-monitoring of glucose tests, non-severe hypoglycaemia, diastolic blood pressure, eGFR and number of people with an amputation.

**Table 1:** Characteristics of the participants

Parameters	Participants			Non-responders (n = 75)
	All (n = 216)	DM1 (n = 107)	DM2 (n = 109)	
Women, n (%)	105 (48.8)	62 (57.9)	43 (39.4)	26 (34.7)*
Age (years)	54.0 ± 17.0	45.9 ± 16.1	62.0 ± 13.7	65.6 ± 15.5*
Duration of diabetes (years)	17.5 ± 11.0	18.7 ± 10.3	16.2 ± 11.5	19.8 ± 13.4
BMI (kg/m^2^)	29.9 ± 7.3	26.6 ± 5.0	33.0 ± 7.7	30.3 ± 8.7
HbA1c				
% mmol/mol	8.7 ± 1.9 71.4 ± 20.3	8.4 ± 1.6 67.8 ± 17.9	8.9 ± 1.9 74.3 ± 20.8	9.1 ± 2.7 76.0 ± 24.8
HbA1c, DCCT adjusted				
% mmol/mol	7.9 ± 1.7 63.1 ± 18.5	7.6 ± 1.5 59.6 ± 16.3	8.2 ± 1.8 66.6 ± 19.8	8.2 ± 2.1 67.0 ± 22.6
Diabetes therapy, n (%)				
Diet OAD/GLP1 agonists Insulin	7 (3.2) 7 (3.2) 202 (93.6)	0 (0) 0 (0) 107 (100)	7 (6.4) 7 (6.4) 95 (87.2)	2 (2.7) 9 (12.0) 64 (85.3)
Insulin therapy, n (%)				
No insulin Basal insulin Premixed insulin Short acting insulin (+ basal insulin) Insulin pump	14 (6.5) 7 (3.2) 9 (4.2) 150 (69.4) 36 (16.7)	0 (0) 0 (0) 2 (1.9) 70 (65.4) 35 (32.7)	14 (12.8) 7 (6.4) 7 (6.4) 80 (73.5) 1 (0.9)	11 (14.7)* 3 (4.0)* 4 (5.3)* 53 (70.7)* 4 (5.3)*
Insulin dosage				
Total (IU/day) Basal (IU/day)	62.0 ± 46.3 24.8 ± 22.0	47.0 ± 26.2 22.5 ± 14.0	78.9 ± 57.1 27.4 ± 28.3	75.3 ± 68.3 25.1 ± 24.8
Number of insulin injections per day	3.4 ± 1.6	3.3 ± 1.9	3.6 ± 1.2	3.6 ± 1.2
Number of self-monitoring of glucose tests per day (blood, CGM, FGM)	3.7 ± 3.0	4.5 ± 3.8	3.2 ± 1.6	2.8 ± 1.7*
Frequency of non-severe hypoglycaemia per week	1.6 ± 2.9	2.8 ± 3.7	0.4 ± 0.9	0.7 ± 1.4*
Severe hypoglycaemia past 12 months				
Frequency Number of events (people with an event)	0.08 ± 0.4 18 (12)	0.09 ± 0.4 10 (7)	0.07 ± 0.4 8 (5)	0.04 ± 0.2 0 (0)
Ketoacidosis past 12 months				
Frequency Number of events (people with an event)	0.03 ± 0.2 7 (5)	0.07 ± 0.3 7 (5)	0 0 (0)	0.03 ± 0.2 0 (0)
Blood pressure				
Systolic (mmHg) Diastolic (mmHg) Pulse (bpm)	136.3 ± 18.6 80.7 ± 12.8 76.5 ± 11.8	134.1 ± 15.9 82.7 ± 11.2 86.0 ± 9.4	138.4 ± 20.7 78.8 ± 14.0 77.0 ± 13.8	131.9 ± 17.7 75.8 ± 13.1* 74.6 ± 12.2
eGFR (ml/min)	73.3 ± 28.2	85.0 ± 26.5	61.9 ± 25.0	59.3 ± 29.1*
eGFR < 60 ml/min, n (%)				
≥ 60 ml/min 30–59 ml/min < 30 ml/min	143 (66.2) 62 (28.7) 11 (5.1)	90 (84.1) 11 (10.3) 6 (5.6)	53 (48.6) 51 (46.8) 5 (4.6)	29 (38.7)* 34 (45.3)* 12 (16.0)*
Albumin/g creatinine (mg/g)	246.2 ± 906.8	117.1 ± 312.7	371.6 ± 1225.6	168.1 ± 389.0
Albumin/g creatinine, n (%)				
< 30 30–300 mg/g 301–3000 mg/g > 3000 mg/g	138 (63.9) 52 (24.1) 21 (9.7) 5 (2.3)	73 (68.3) 24 (22.4) 10 (9.3) 0 (0)	65 (59.6) 28 (25.7) 11 (10.1) 5 (4.6)	27 (36.0) 35 (46.7) 7 (9.3) 0 (0)
Polyneuropathy, n (%)	93 (43.1)	27 (25.2)	66 (60.6)	42 (56.0)
Severe diabetes-related complications, n (%)				
Amputation Dialysis Blindness	3 (1.4) 1 (0.5) 0 (0)	1 (0.9) 1 (0.9) 0 (0)	2 (1.8) 0 (0) 0 (0)	6 (8.0)* 3 (4.0) 0 (0)

*BMI* body mass index, *CGM* continuous glucose monitoring, *DM1* diabetes mellitus type 1, *DM2* diabetes mellitus type 2, *eGFR* estimated glomerular filtration rate, *FGM* flash glucose monitoring, *GLP1* glucagon like peptide 1, *IU* insulin units, *OAD* oral antidiabetic drugs

^*^p-value < 0.05 between all participants and dropout

### Diabetes-related distress

The PAID questionnaire was completed by 214 of 216 participants. Fifty-one of 214 individuals (23.8%) scored ≥ 40 points in the PAID questionnaire indicating that these people had high diabetes-related distress. The mean PAID score of all participants was 28.1 ± 17.5 (range 0–81.25).

The items scoring highest were (in descending order): “worries about the future and serious complications” (mean PAID score 2.1 ± 1.2), “feelings of guilt for suboptimal diabetes management” (1.7 ± 1.2) and “worries about hypoglycaemia” (1.6 ± 1.3) (Table 2). The item scoring lowest was “dissatisfaction with support from family and friends” (0.5 ± 0.9). The differences between people with DM1 and DM2 are shown in Table 2.

**Table 2:** Mean score (range 0–4) of each item of the PAID questionnaire

Item	All (n = 214)	DM1 (n = 107)	DM2 (n = 107)	p-value*
1. Not having clear and concrete goals for your care?	1.4 ± 1.3	1.0 ± 1.1	1.8 ± 1.3	**< 0.001**
2. Feeling discouraged with your diabetes treatment plan?	1.3 ± 1.2	1.1 ± 1.1	1.5 ± 1.3	**0.013**
3. Feeling scared when you think about living with diabetes?	1.1 ± .1.2	1.1 ± 1.1	1.1 ± 1.2	0.757
4. Uncomfortable social situations related to your diabetes care?	1.0 ± 1.1	1.1 ± 1.1	0.9 ± 1.0	0.152
5. Feelings of deprivation regarding food and meals?	1.2 ± 1.2	1.1 ± 1.1	1.4 ± 1.2	0.107
6. Feeling depressed when you think about living with diabetes?	0.9 ± 1.1	0.9 ± 1.2	0.8 ± 1.0	0.321
7. Not knowing if your mood or feelings are related to your diabetes?	1.1 ± 1.1	1.2 ± 1.1	1.0 ± 1.0	0.185
8. Feeling overwhelmed by your diabetes?	0.9 ± 1.0	1.0 ± 1.0	0.9 ± 1.0	0.309
9. Worrying about low blood sugar reactions?	1.6 ± 1.3	1.9 ± 1.3	1.3 ± 1.2	**0.003**
10. Feeling angry when you think about living with diabetes?	0.9 ± 1.0	0.9 ± 0.9	0.9 ± 1.2	0.806
11. Feeling constantly concerned about food and eating?	1.1 ± 1.1	1.1 ± 1.1	1.2 ± 1.1	0.323
12. Worrying about the future and the possibility of serious complications?	2.1 ± 1.2	2.1 ± 1.2	2.1 ± 1.1	0.951
13. Feeling of guilt or anxiety when you get off track with your diabetes management?	1.7 ± 1.2	1.8 ± 1.2	1.6 ± 1.2	0.204
14. Not “accepting” your diabetes?	0.7 ± 1.0	0.8 ± 1.1	0.5 ± 0.9	**0.031**
15. Feeling dissatisfied with your diabetes physician?	0.6 ± 1.0	0.6 ± 0.9	0.6 ± 1.1	0.720
16. Feeling that diabetes is taking up too much of your mental and physical energy every day?	1.2 ± 1.2	1.3 ± 1.2	1.0 ± 1.1	**0.045**
17. Feeling alone with your diabetes?	0.7 ± 1.0	0.7 ± 1.0	0.7 ± 1.0	0.882
18. Feeling that your friends and family are not supportive of your diabetes management efforts?	0.5 ± 0.9	0.5 ± 0.8	0.5 ± 1.0	0.772
19. Coping with complications of diabetes?	1.3 ± 1.2	1.2 ± 1.2	1.5 ± 1.2	0.054
20. Feeling “burned out” by the constant effort needed to manage diabetes?	1.1 ± 1.1	1.2 ± 1.2	1.0 ± 1.1	0.102
Total PAID score (range 0–100)	28.1 ± 17.5	28.1 ± 17.4	26.2 ± 16.9	0.532

*DM1* diabetes mellitus type 1, *DM2* diabetes mellitus type 2

^*^Between DM1 and DM2

Bold values indicate that the significant difference

The number of participants with a PAID score ≥ 40 (25.2% vs. 22.4%, p = 0.749) as well as mean PAID score (28.1 ± 17.4 vs. 26.2 ± 16.9, p = 0.532) was comparable between individuals with DM1 and DM2. Individuals with DM2 on insulin scored higher (n = 93, 27.8 ± 17.6) than study participants without insulin did (n = 14, 18.7 ± 8.5, p = 0.004). However, PAID score was not different between the types of insulin therapy, equally in people with DM1 (premixed insulin: 30.0 ± 19.4 short acting/basal insulin: 30.2 ± 17.5, insulin pump: 23.8 ± 16.9) and DM2 (basal insulin: 29.8 ± 15.5, premixed insulin: 25.2 ± 16.2, short acting insulin: 29.6 ± 18.2, short acting/basal insulin: 27.3 ± 18.2, insulin pump: 28.8). In addition, number of insulin injections per day did not differ between insulin-treated participants with PAID score ≥ 40 (n = 51, 3.8 ± 1.5) and < 40 (n = 149, 3.3 ± 1.7; p = 0.093). Individuals with DM1 treated with a system for continuous glucose monitoring (n = 50, 33.1 ± 18.8) scored higher than participants without such system (n = 32, 20.6 ± 13.3, p = 0.001).

HbA1c was not correlated with the PAID score in both, DM1 (r = 0.040, p = 0.684) and DM2 (r = − 0.024, p = 0.804). Participants with DM2 who had incurred a severe hypoglycaemia/last 12 months scored higher (n = 5, 43.0 ± 20.4) than people without such an event (n = 102, 25.1 ± 16.5, p = 0.026). The frequency of non-severe hypoglycaemic events was not associated with the PAID score neither in people with DM1 nor DM2.

There were no associations between PAID score and dialysis, amputation or presence of diabetic polyneuropathy neither in DM1 nor in DM2. In addition, PAID score was not correlated with eGFR or albumin/g creatinine ratio.

### Wellbeing, quality of life and satisfaction with diabetes treatment

Questionnaires were completed as follows: WHO5 n = 196, ADDQoL n = 148, DTSQs n = 210. Mean WHO-5 Well-being Index was 14.1 ± 6.3, mean ADDQoL score − 1.7 ± 1.4 and mean DTSQs score 24.9 ± 6.7 with no statistically significant difference between people with DM1 and DM2.

Sixty-nine of 196 participants (35.2%) scored < 13 points in the WHO-5 questionnaire thus potentially suffering from depression (DM1: 35.1% vs. DM2: 35.4%, p = 0.542).

The PAID score was negatively correlated with the WHO-5 Well-being Index (r = − 0.386, p < 0.001), the ADDQoL (r = − 0.525, p < 0.001) as well as the DTSQs score (r = − 0.305, p < 0.001).

People with DM1 and insulin pump therapy reported higher treatment satisfaction (DTSQs score: 26.8 ± 6.4) than individuals with intensified insulin therapy with short acting/basal insulin (23.4 ± 6.3, p = 0.012). In participants with DM2, quality of life was different between the types of insulin therapy (basal insulin: − 0.5 ± 0.5, premixed insulin: − 1.8 ± 0.4, short acting insulin: − 2.1 ± 1.7, short acting/basal insulin: − 1.5 ± 1.2, insulin pump: − 2.4).

## Discussion

The primary aim of this study was to assess diabetes-related distress and to investigate its association with metabolic control in people with DM1 and DM2 in an inpatient setting. Our expectation as well as hypothesis was that diabetes-related distress is higher in inpatients than in patients treated at primary/secondary care level in Germany. In contrast to outpatients, patients with DM1 as well as DM2 treated in hospital suffered more frequently from diabetes-related distress. Approximately 24% of our inpatients showed high diabetes-related distress (as defined by PAID ≥ 40).

The German national guideline “Psychosocial and Diabetes” mentions a high prevalence of depressive disorders, especially diabetes-related distress, effecting an estimated 25% of people with diabetes [1]. This statement is based for example on the study by Hermanns et al. (2006) reporting a prevalence of 30% [7]. In contrast, other studies show a considerably lower prevalence of diabetes-related distress of < 10% [5, 12, 13]. There are many possible reasons for conflicting data. Hereby the investigation setting is one of the main factors. E.g., a cross-sectional Danish survey of about 2400 adult outpatients with DM1 showed a prevalence of 9.8% [21]. In Germany, investigations in an outpatient setting similarly showed a low prevalence of diabetes-related distress of 1.2% and 8.9%, respectively on primary as well as secondary care level [5, 13]. In contrast, the study by Hermanns et al. (2006) with a prevalence of 30% was conducted in an inpatient setting [7], thus making the prevalence comparable to this study with its 24%. Guideline should consider this and be amended accordingly. In addition, care level (outpatients and inpatients) is not the only reason. There may also be differences in distress levels between countries due to different healthcare systems. A multinational, cross-sectional survey—the DAWN2 survey—showed different prevalence of high diabetes-related distress around the world (about 20–70%) [22].

Since the prevalence of distress increases with higher level of care, it needs to be cogitated which reasons might be responsible for this finding. Compared to the primary or even secondary care level, inpatients were treated more often with insulin, had higher HbA1c levels and more diabetes-related problems, etc. (Table 3). The present study showed that treatment with insulin is associated with higher diabetes-related distress. Individuals with DM2 on insulin scored 9.1 points higher (on a scale of maximum 100 point) than patients without insulin. Most people with diabetes at primary care level are treated without insulin therapy, even often with no antidiabetic drug [23]. The low burden of the disease at that stage could explain the low level of distress of 1.2% [13]. Climbing up the levels of care the diabetes therapy is becoming more and more complex (more insulin injections and glucose test per day as well as more diabetes technology). The disease itself as well as therapeutic procedures obviously place a strain on individuals with diabetes. We found that people with DM1 treated with a system for continuous glucose monitoring scored 12.5 points higher than patients without such system did. This indicates that patients with such a system have more problems with diabetes therapy and therefore suffer more from distress or may be stressed by the device itself.

**Table 3:** Diabetes-related distress dependent on care level

Setting	Number of participants	Mean age (years)	Insulin treatment (% participants)	Mean HbA1c (%)*	Mean PAID score	People with PAID score ≥ 40 points (%)
Outpatients, primary care [13]	All 345 (DM1 = 9; DM2 = 336)	DM1: 59.8 DM2: 72.3	All: 33.6 (DM1: 100; DM2: 31.8)	DM1: 7.5 DM2: 6.4	All: **3.9** (DM1: 6.8; DM2: 3.8)	All: **1.2** (DM1: 0; DM2: 1.2)
Outpatients, secondary care [5]	All 783 (DM1 = 191; DM2 = 592)	All: 63.7 (DM1: 54.5; DM2: 66.6)	All: 76.6 (DM1: 100; DM2: 69.3)	DM1: 7.2 DM2: 7.0	All: **17.1** (DM1: 17.8; DM2: 16.8)	All: **8.9** (DM1: 8.8; DM2: 9.0)
Inpatients [present study]	All 216 (DM1 = 107; DM2 = 109)	All: 54.0 (DM1: 45.9; DM2: 62.0)	All: 93.6 (DM1: 100; DM2: 87.2)	All: 7.9 DM1: 7.6 DM2: 8.2	All: **28.1** (DM1: 28.1; DM2: 26.2)	All: **23.8** (DM1: 25.2; DM2: 22.4)

*DM1* diabetes mellitus type 1, *DM2* diabetes mellitus type 2

^*^DCCT adjusted

Bold values indiacte that the better readability

However, regardless of level of care or setting, the greatest fear of diabetes patients is “worries about the future and serious complications” [5, 13] though the frequency of serious complications (e.g. blindness, dialysis or amputation) is very low in Germany [23]. This was also shown in a cross-sectional study [24]. People with DM1 and DM2 overestimated their risk regarding long-term complications by far. Obviously proper information regarding the actual risk of diabetes-related complications is lacking.

Previous studies showed that those with psychological disorders, such as depression or high diabetes-related distress, are more often female in the general population [25] as well as in people with diabetes [5, 9]. In the present study, no difference between women and men was present which could imply that problems with the diabetes therapy itself are the reason for the increased distress.

Apart from the setting of the investigation, also the measurement tool used is responsible for different results of studies. The DAWN2 survey showed a prevalence of high distress of 25% for people recruited in Germany [22]. This survey assessed diabetes-related distress with the PAID-5 scale, a short form of the PAID questionnaire consisting only of 5 questions. This could possibly be responsible for the different data. Furthermore, it is plausible that diabetes-related distress is interdependent with many variables such as actual well-being or living conditions. In a population-based, prospective trial 506 participants with DM2 were assessed three times over 18 months for different psychological outcomes, including the Diabetes Distress Scale, which also measures distress [3]. The overall prevalence of distress was 29.2% over 18 months at any of the three times (baseline, 9 months follow-up and 18 months follow-up) whereas only 6.4% of the responders had high distress for all three times.

As secondary outcome, our study showed a negative correlation between PAID score and Well-being Index, ADDQoL score as well as DTSQs score. That indicates that high diabetes-related distress can negatively affect well-being, quality of life and treatment satisfaction (or vice versa). One trigger for distress in people with diabetes could be the perceived limitations in quality of life, for example, due to high daily frequency of insulin injections and glucose tests. However, this affects only a small number of patients with DM2 (especially at higher care levels) and thus dilutes in samples with low number of patients on insulin. Therefore, routine screening for depression is important and should be done on a regular basis. Taking into account the different prevalence rates, screening for diabetes-related distress is of particular importance in inpatients. In outpatients, due to the low prevalence especially on primary care level, screening may be less reasonable [13].

A strength of our trial is that the participants are well characterised. All used questionnaires are evaluated and validated. However, our investigation has also some limitations. Limitations are the significant differences between the study participants and non-responders regarding baseline characteristics as well as the study design, which does not allow any causal relationships.

## Conclusions

Patients with diabetes type 1 as well as type 2 treated in hospital for problems with diabetes therapy suffer frequently from diabetes-related distress (~ 24%) in Germany. Guidelines should consider that diabetes related distress increases markedly with the level of care and is low on primary care level.

## Data Availability

All data generated or analysed during this study are included in this published article.

## Abbreviations

ADDQoL: Audit of diabetes dependent quality of life
BMI: Body mass index
DM1: Diabetes mellitus type 1
DM2: Diabetes mellitus type 2
DTSQ: Diabetes treatment satisfaction questionnaire
eGFR: Estimated glomerular filtration rate
PAID: Problem area in diabetes
WHO: World Health Organization

## References

[1] Kulzer B AC, Herpertz S, Kruse J, Lange K, Lederbogen F, Petrak F. S2-Guideline “Psychosocial and Diabetes”. 2013. www.deutsche-diabetes-gesellschaft.de. Last accessed 13 May 2020.

[2] Gonzalez JS, Shreck E, Psaros C, Safren SA (2015). Distress and type 2 diabetes-treatment adherence: a mediating role for perceived control. Health Psycholog Off J Div Health Psycholog Am Psycholog Assoc.

[3] Fisher L, Skaff MM, Mullan JT, Arean P, Glasgow R, Masharani U (2008). A longitudinal study of affective and anxiety disorders, depressive affect and diabetes distress in adults with Type 2 diabetes. Diabet Med.

[4] Reddy J, Wilhelm K, Campbell L (2013). Putting PAID to diabetes-related distress: the potential utility of the problem areas in diabetes (PAID) scale in patients with diabetes. Psychosomatics.

[5] Kuniss N, Kramer G, Müller N, Kloos C, Lehmann T, Lorkowski S, Wolf G, Müller UA (2016). Diabetes-related burden and distress is low in people with diabetes at outpatient tertiary care level. Exp Clin Endocrinol Diabetes.

[6] Pouwer F, Skinner TC, Pibernik-Okanovic M, Beekman AT, Cradock S, Szabo S (2005). Serious diabetes-specific emotional problems and depression in a Croatian-Dutch-English Survey from the European Depression in Diabetes [EDID] Research Consortium. Diabetes Res Clin Pract.

[7] Hermanns N, Kulzer B, Krichbaum M, Kubiak T, Haak T (2006). How to screen for depression and emotional problems in patients with diabetes: comparison of screening characteristics of depression questionnaires, measurement of diabetes-specific emotional problems and standard clinical assessment. Diabetologia.

[8] American Diabetes Association (2019). Standards of medical care in diabetes—2019. Diabetes Care.

[9] Snoek FJ, Pouwer F, Welch GW, Polonsky WH (2000). Diabetes-related emotional distress in Dutch and U.S. diabetic patients: cross-cultural validity of the problem areas in diabetes scale. Diabetes Care.

[10] Welch GW, Jacobson AM, Polonsky WH (1997). The problem areas in diabetes scale. An evaluation of its clinical utility. Diabetes Care.

[11] World Health Organization info package (1998). Mastering depression in primary care.

[12] Stoop CH, Nefs G, Pop VJ (2014). Diabetes-specific emotional distress in people with Type 2 diabetes: a comparison between primary and secondary care. Diabet Med.

[13] Kuniss N, Rechtacek T, Kloos C, Müller UA, Roth J, Burghardt K, Kramer G (2017). Diabetes-related burden and distress in people with diabetes mellitus at primary care level in Germany. Acta Diabetol.

[14] Bradley C, Todd C, Gorton T, Symonds E, Martin A, Plowright R (1999). The development of an individualized questionnaire measure of perceived impact of diabetes on quality of life: the ADDQoL. Qual Life Res Intern J Qual Life Aspects Treat Care Rehabil.

[15] Kubiak T, Hermanns N, Krichbaum M, Kulzer B, Haak T. Erfassung der diabetesbezogenen Therapiezufriedenheit mit der deutschsprachigen Fassung des Diabetes Treatment Satisfaction Questionnaire (DTSQ) psychometrische Eigenschaften und Validierung. Bericht über die 38. Jahrestagung der Deutschen Diabetesgesellschaft in Bremen. Diabetes und Stoffwechsel 2003;12(1):56.

[16] Bradley C, editors. The diabetes treatment satisfaction questionnaire: DTSQ. Handbook of psychology and diabetes: a guide to psychological measurements in diabetes research and practice. Chur, Switzerland: Harwood Academic Publishers; 1994.

[17] Schumann M. Electronic medical information system for long-term documentation of chronic diseases (EMIL). 2013. http://cleverdoku.de. Last accessed 02 Sept 2015.

[18] Seaquist ER, Anderson J, Childs B, Cryer P, Dagogo-Jack S, Fish L (2013). Hypoglycemia and diabetes: a report of a workgroup of the American Diabetes Association and the Endocrine Society. Diabetes Care.

[19] German Diabetes Association (DDG). Guidelines for recognition of a treatment institution—Certified Diabetes Center DDG (valid from 15.04.2020). 2015. http://www.deutsche-diabetes-gesellschaft.de/fileadmin/Redakteur/Zertifizierung/Basisanerkennung/Richtlinie_Zert_Diabeteszentrum_DDG_2015.pdf. Last accessed 13 May 2020.

[20] The Diabetes Control and Complications Trial Research Group (1993). The effect of intensive treatment of diabetes on the development and progression of long-term complications in insulin-dependent diabetes mellitus. NEJM.

[21] Joensen LE, Almdal TP, Willaing I (2013). Type 1 diabetes and living without a partner: psychological and social aspects, self-management behaviour, and glycaemic control. Diabetes Res Clin Pract.

[22] Nicolucci A, Kovacs Burns K, Holt RI, Comaschi M, Hermanns N, Ishii H (2013). Diabetes attitudes, wishes and needs second study (DAWN2): cross-national benchmarking of diabetes-related psychosocial outcomes for people with diabetes. Diabet Med.

[23] Groos S, Kretschmann J, Macare C, Weber A, Hagen B. Quality Assurance Report 2018 Disease-Management-Programmes in Northrhine. Available at https://www.kvno.de/downloads/quali/qualbe_dmp18.pdf. Last accessed 13 May 2020.

[24] Arend F, Müller UA, Schmitt A, Voigt M, Kuniss N (2019). Overestimation of risk and increased fear of long-term complications of diabetes in people with Type 1 and 2 diabetes. Exp Clin Endocrinol Diabetes.

[25] Busch MA, Maske UE, Ryl L, Schlack R, Hapke U (2013). Prevalence of depressive symptoms and diagnosed depression among adults in Germany: results of the German Health Interview and Examination Survey for Adults (DEGS1). Bundesgesundheitsblatt Gesundheitsforschung Gesundheitsschutz.

